# Dose-Escalated Radiotherapy for Primary Tracheobronchial Adenoid Cystic Carcinoma

**DOI:** 10.3390/cancers16112127

**Published:** 2024-06-03

**Authors:** Jeong Ha Lee, Jeong Yun Jang, Jae Myoung Noh, Kyungmi Yang, Hongryull Pyo

**Affiliations:** 1 Department of Radiation Oncology, Samsung Medical Center, Sungkyunkwan University School of Medicine, Seoul 06351, Republic of Korea; jeongha.lee@samsung.com (J.H.L.); jeongyun.jang@kuh.ac.kr (J.Y.J.); rodrno@skku.edu (J.M.N.); 2 Department of Radiation Oncology, Konkuk University Medical Center, Konkuk University School of Medicine, Seoul 06351, Republic of Korea

**Keywords:** tracheal cancer, adenoid cystic carcinoma, radiotherapy

## Abstract

**Simple Summary:**

The optimal radiotherapy (RT) dose for tracheobronchial adenoid cystic carcinoma (ACC) remains unclear due to its scarcity. This retrospective study evaluated the efficacy of dose-escalated RT for primary tracheobronchial ACC by dividing patients into groups of either low (<70.0 Gy EQD2) or high (≥70.0 Gy EQD2) RT doses. In the definitive RT group, the high-dose group showed better local control and survival rates compared to the low-dose group. The treatment related toxicities were the trachea or main bronchus stenosis. Dose-escalated RT may be effective for the definitive treatment of tracheobronchial ACC.

**Abstract:**

Primary tracheobronchial adenoid cystic carcinoma (ACC) is a rare malignancy, so the optimal radiotherapy (RT) dose remains unestablished. We aimed to evaluate the effectiveness of dose-escalated RT for primary tracheobronchial ACC. We retrospectively reviewed 48 patients who had undergone definitive or postoperative RT. Patients classified into the low- and high-dose groups received RT doses <70.0 and ≥70.0 Gy in EQD2, respectively. The primary endpoint was freedom from local progression (FFLP) and overall survival (OS). Throughout the follow-up period, seven patients (14.6%) experienced local progression, while 31 (64.6%) exhibited distant metastasis, most commonly in the lungs. In total, the 5-year FFLP and OS rates were 85.7 and 84.7%, respectively. Multivariate analysis revealed that regional lymph node metastasis at diagnosis and receipt of definitive RT were associated with poorer OS. In the subgroup analysis, the definitive RT group had a 5-year FFLP rate of 33.3 and 78.2% in the low- and high-dose groups (*p* = 0.065), whereas 5-year OS rates were 66.7 and 79.0%, respectively (*p* = 0.022). Four patients (8.3%) experienced Grade 3 toxicity with tracheal or main bronchus stenosis. Dose-escalated RT with conventional fractionation may be effective in patients with tracheobronchial ACC, especially for a definitive aim.

## 1. Introduction

Tracheobronchial adenoid cystic carcinoma (ACC) is a rare malignancy accounting for <0.2% of all lung cancers [1]. Originating from the submucosal gland of the tracheobronchial tree, tracheobronchial ACC is classified as a low-grade malignancy; however, frequent distant metastasis and late local relapse have been documented [2].

Although the surgical resection of the tumor is considered the optimal treatment approach, some patients are deemed unresectable owing to factors such as tumor size, involvement of adjacent organs with locally advanced tumors, presence of distant metastasis, or poor general condition [3]. Furthermore, as revealed in one surgical case series, >60% of patients had grossly or microscopically positive tumors after curative surgery, highlighting the challenges in attaining R0 resection and warranting the need for adjuvant treatment in >50% of these patients [4]. Accordingly, radiotherapy (RT) has been proposed and utilized as an alternative approach for patients with unresectable tumors or as an adjuvant treatment for those who could not achieve sufficient margin after curative surgery [5].

However, there is a scarcity of relevant reports owing to the rarity of the disease; hence, the optimal method and dose of RT in patients with tracheobronchial ACC remains unestablished. High-dose RT has been shown to improve local control [6,7]. In contrast, cases of fibrosis and stenosis have been documented after high-dose irradiation to the trachea, potentially resulting in severe dyspnea and respiratory failure [8]. These inconclusive findings regarding using high-dose RT for tracheal ACC complicate the decision-making process for radiation oncologists. Furthermore, most reports of dose escalation involved small sample sizes or were limited to case reports, with a lack of clear evidence from large studies. Therefore, herein, we aimed to evaluate the effectiveness and safety of dose-escalated RT as a definitive or adjuvant treatment for patients with tracheobronchial ACC.

## 2. Material and Methods

### 2.1. Patients

**Figure 1:**
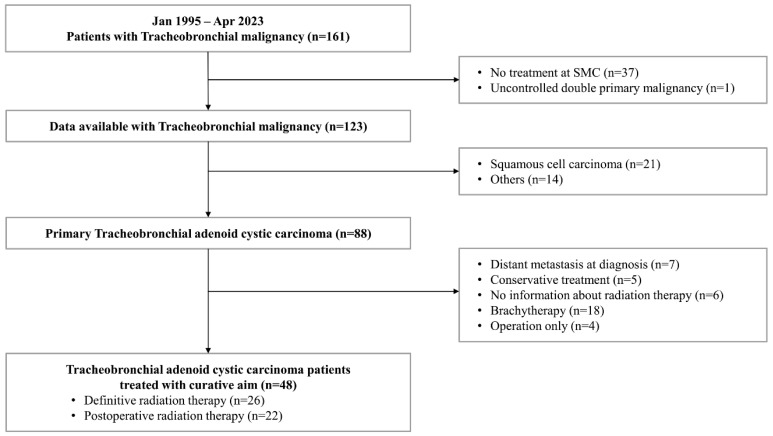
Flow diagram for selecting patients with tracheobronchial adenoid cystic carcinoma for radiation therapy.

We retrospectively reviewed the medical records of 161 patients with tracheobronchial malignancy who visited Samsung Medical Center from January 1995 to April 2023. We included patients who (1) were diagnosed histologically with tracheobronchial ACC, (2) did not have distant metastasis at diagnosis, and (3) were treated with external beam radiotherapy (EBRT) either as a definitive or postoperative aim in our center. Finally, a total of 48 patients were included in the analysis (Figure 1). Tumor location and size were evaluated by bronchoscopy and chest computed tomography (CT) images, and staging was determined using Bhattacharyya’s staging system [9].

### 2.2. Treatments

Surgery was performed when complete resection was feasible, considering the patient’s performance status, comorbidities, and the primary tumor location and size, with the decision reached either by a skilled thoracic surgeon or during a multidisciplinary team meeting. Patients who could not achieve R0 resection or had close resection margin underwent postoperative RT (PORT) 4–6 weeks after surgery, whereas those with tumors not eligible for surgery received definitive RT. No patient received chemotherapy.

For RT, the gross tumor volume (GTV) was delineated to include all visible tumors, including metastatic lymph nodes observed in bronchoscopy or chest CT; for PORT cases, the tumor bed was delineated based on preoperative images, surgical records, and pathology reports. The clinical target volume (CTV) was contoured with a cranial-caudal margin of 5–10 mm and a circumferential margin of 5–7 mm from the GTV or tumor bed. Elective nodal irradiation was not performed. The planning target volume (PTV) was created by adding a 5–7 mm margin to the CTV.

The RT doses were converted to an equivalent dose at 2.0 Gy (EQD2) using an α/β ratio of 2 [10]. The median EQD2 was 74.0 Gy (range, 56.3–83.5 Gy), delivered at a median of 2.0 Gy per fraction (Gy/fx) (range 1.8–3.0 Gy). Patients were classified into two dose groups based on the EQD2: the low- and high-dose groups comprised those who received RT doses <70.0 and ≥70.0 Gy, respectively. A detailed dose scheme and the number of patients for each group are shown in Table 1. Except for one patient who received two-dimensional RT, all patients were planned with a three-dimensional conformal radiotherapy or intensity-modulated RT (IMRT).

### 2.3. Response Evaluation and Outcomes

Patients underwent regular follow-up, including chest CT or bronchoscopy (with or without biopsy), at 3-month intervals for the first 2 years and every 6 months thereafter. The tumor response was evaluated 3 months after end of RT using the Response Evaluation Criteria in Solid Tumors version 1.1 [11].

Freedom from local progression (FFLP) was defined as the duration between treatment initiation to local tumor progression in the trachea and/or main bronchus, specifically within the PTV margin; overall survival (OS) was defined as the duration from treatment initiation to mortality. Local progression was confirmed when follow-up chest CT or bronchoscopy revealed endobronchial narrowing or tumor growth with enhancement, which was subsequently confirmed via biopsy or increased FDG uptake in positron emission tomography-CT. Treatment-related toxicity was evaluated based on the Common Terminology Criteria for Adverse Events version 5.0.

### 2.4. Statistical Analyses

The chi-square test and t-test were performed to compare the distribution of patient characteristics in each treatment group. FFLP and OS were calculated using the Kaplan–Meier method, and the log-rank test was used to compare the differences between the groups. Univariate and multivariate analyses were performed using Cox regression analysis. A *p*-value < 0.05 was considered statistically significant. Data analyses were performed using SPSS ver. 23.0 (IBM Corp., Armonk, NY, USA).

## 3. Results

### 3.1. Patient’s Characteristics

Forty-eight patients were included in the final analysis, and their characteristics are summarized in Table S1. The median age was 50 years (range, 24–76 years), and 23 patients (47.9%) were male. The mean tumor size was 2.5 cm (standard deviation, 0.8 cm). Twenty-two patients (45.8%) underwent PORT, and 26 (54.2%) received definitive RT. Regarding the RT dose, 21 patients from the low-dose group (43.8%) received a median EQD2 of 60.0 Gy (range, 56.3–69.3 Gy). The high-dose group comprised 27 (56.3%) who received a median of EQD2 75.0 Gy (range, 70.0–82.5 Gy).

### 3.2. Treatment Response and Pattern of Failure

Three months after treatment completion, no patients experienced disease progression. Among the patients who underwent definitive RT, one patient (3.8%) achieved a complete response, 20 (76.9%) showed a partial response, and five (19.2%) had the stable disease.

Treatment outcomes and patterns of failures are summarized in Table 2. During a median follow-up period of 62.2 months (interquartile range, 27.2–110.8 months), seven patients (14.6%) experienced local progression, whereas 31 patients (64.6%) presented with distant metastasis. Local progression was developed at a median of 44.4 months (range, 9.8–62.9 months) from RT initiation. Among the patients with local progression, three exhibited subsequent distant progression, whereas the other four had distant metastasis prior to the local progression, all involving the lung as the metastasis site. The most common sites for distant metastasis were the lung, bone, liver, and mediastinal lymph nodes, in that order. The most frequent first failure site was the lung (45.8%), followed by local progression of the primary tumor at the trachea or main bronchus (6.3%). Subsequent treatments after local progression and distant metastasis are shown in Table S2.

### 3.3. Oncologic Outcomes

Considering all patients, the 5-year FFLP and OS rates were 85.7 and 84.7%, respectively. Regarding RT, the 5-year FFLP rate for the definitive RT and PORT was 69.7 and 100% (*p* = 0.001), while the 5-year OS was 76.7 and 90.5%, respectively (*p* = 0.005).

In the univariate analysis, no factor was associated with FFLP (Table S3). However, positive lymph node at diagnosis (hazard ratio [HR], 8.66; 95% confidence interval [CI], 2.12–35.43, *p* = 0.003) and receipt of definitive RT (HR, 6.41; 95% CI, 1.79–23.00, *p* = 0.004) were identified as prognostic factors associated with inferior OS in the multivariate analysis (Table S4).

### 3.4. Subgroup Analysis Regarding RT and RT-Dose Group

To evaluate the effectiveness of dose escalation in groups that underwent surgery and those that did not, we conducted a subgroup analysis within each group based on RT doses (Table S5). Considering the PORT group, 17 (77.3%) and 5 (22.5%) patients were assigned to the low- and high-dose groups, respectively. In the definitive RT group, four (15.4%) and 22 (84.6%) patients were present in the low- and high-dose groups, respectively.

**Figure 2:**
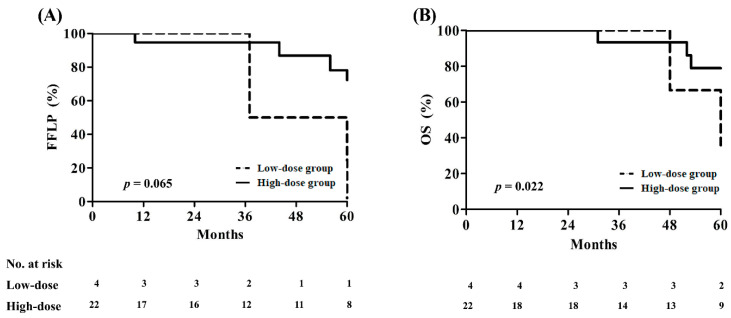
(**A**) Freedom from local progression and (**B**) overall survival in the definitive radiotherapy group by dose.

Pattern of failures by the aim of radiotherapy and dose group are shown in Table S6. In the PORT group, no patient experienced local progression, and the 5-year OS rates in the low- and high-dose groups were 88.2 and 100%, respectively (*p* = 0.230). In the definitive RT group, seven patients experienced local recurrence, with two belonging to the low-dose group and five to the high-dose group; the 5-year FFLP rates were 33.3 and 78.2% in the low- and high-dose groups, respectively (*p* = 0.065) (Figure 2A). The 5-year OS rates were 66.7 and 79.0% in the low- and high dose groups, respectively (*p* = 0.022) (Figure 2B). Table 3 summarizes the characteristics of patients experiencing local progression.

### 3.5. Treatment-Related Complications

Four patients (8.3%), all belonging to the definitive RT group, experienced Grade 3 treatment-related toxicity with tracheal or main bronchus stenosis requiring repeated bronchoscopy and stent insertion (Table S7). Among them, three (75.0%) patients were treated with 3.0 Gy/fx, and three (75.0%) had tumors involving the main bronchus. No Grade 4 or 5 toxicities were observed.

## 4. Discussion

Considering the potential challenges in surgical resection or the achievement of R0 resection despite surgery, RT is typically required for definitive and postoperative aims, with a recommended dose >60.0 Gy [6,12,13]. Previous retrospective studies examining treatment outcomes for tracheal ACC have revealed a 5-year OS rate ranging from 40–88% [14,15,16,17]. Despite including patients at various stages, utilizing diverse treatment techniques and policies across institutions complicating direct comparisons, our study still demonstrated a 5-year OS rate of 84.7%.

For dose-escalated RT, previous research on head and neck ACC has demonstrated a correlation between higher radiation doses and enhanced disease control, thereby suggesting a dose–response relationship [18,19]. Therefore, several studies have explored the effectiveness of dose-escalated definitive RT in patients with tracheobronchial ACC, and their findings are summarized in Table 4 [6,15,20,21,22,23,24,25,26]. In a study by Je et al., nine patients who underwent definitive RT for primary tracheal ACC were divided into two groups: a low-dose group receiving EBRT alone with a median dose of 66.0 Gy and a high-dose group receiving EBRT followed by a brachytherapy boost with a median dose of 77.1 Gy [15]. The high-dose group demonstrated superior 5-year local progression-free survival (PFS) of 100.0% when compared with 0.0% in the low-dose group (*p* = 0.002); the 5-year OS rates were 83.3 and 33.3% in the high- and low-dose groups, respectively (*p* = 0.036). The study by Zeng et al. involved a total of 32 patients with primary tracheal carcinoma, of whom 10 were diagnosed with ACC; high-dose RT was defined as ≥68.0 Gy. The authors revealed superior survival outcomes in the high-dose group (5-year PFS, 44.4 vs. 22.2%, *p* = 0.067; 5-year OS, 44.4 vs. 13.0%, *p* = 0.044) [7]. Furthermore, in case reports by Millar et al. and Verma et al., dose escalation was performed up to 80.0 Gy, with the authors reporting no evidence of disease for 6 years with tolerable toxicities [6,25]. Collectively, despite the promising outcomes associated with dose escalation, most of the studies were case reports. Additionally, the retrospective studies frequently included heterogeneous groups with various pathologies, did not distinguish PORT and definitive RT, and resulted in analyses of high-dose RT in primary tracheal ACC limited to a small number of patients. Therefore, our study holds significance, considering the substantial number of patients included with tracheobronchial ACC only. With dose-escalated RT, we demonstrated 100% 5-year FFLP and OS rates for patients undergoing PORT, whereas the 5-year FFLP and OS rates for definitive RT were 78.2 and 79.0%. These findings suggest that a radiation EQD2 of ≥70.0 Gy could be considered a primary treatment option for patients with tracheobronchial ACC who are deemed unresectable for several reasons.

Despite the efficacy of dose escalation, the administration of high-dose RT to the central airway has inherent limitations, considering the possibility of tracheal or bronchial stenosis or fibrosis, which necessitates additional interventions, potentially impacting the patient’s quality of life and, in severe cases, poses a threat to their survival [6]. Consequently, several approaches have been suggested to address this issue. Endobronchial brachytherapy is a notable approach frequently employed to administer high doses of radiation in proximity to the primary tumor, applying high-dose rates to the tumor while sparing the surrounding normal tissue [15,22]. However, when a catheter is placed near the bronchial wall where a larger vessel exists, there is a considerable risk of bleeding, as well as potential late toxicities such as fistula formation, hemoptysis, tracheomalacia, and bronchial stenosis [27,28,29,30]. Furthermore, the procedures and outcomes of brachytherapy can vary substantially depending on the operator, and there may be substantial intra- or inter-fraction discrepancies and errors, rendering it less reproducible [30]. Other approaches involve using EBRT with a simultaneous integrated boost, delivering distinct doses to each area, or the IMRT technique, which improves target conformality and facilitates more accurate radiation delivery. These advancements may improve the feasibility of administering a more selective and higher dose to the primary tumor. Moreover, considering the distinct physical characteristics of particle-beam therapy, including the Bragg peak known for reduced lateral penumbra and rapid dose fall-off, this technique is anticipated to effectively serve as a more precise approach for delivering concentrated doses exclusively to specific areas [31,32]. In one case report, combining a photon beam with a boost of proton beam therapy was utilized to deliver a dose of 80.0 Gy, resulting in no evidence of disease for 11 months, along with the absence of severe complications [6]. Furthermore, Chen et al. employed carbon ion beam therapy at a dose ranging from 72.6–85.8 GyE; the authors reported no Grade ≥ 3 complications [23]. In our study, two patients received intensity modulated proton therapy at a dose of 74.0 GyE, and both maintained a disease-free status to date while exhibiting tolerable toxicity.

The low α/β ratio in ACC, estimated to be approximately 2 in recent studies, suggests the potential for improved treatment outcomes with hypofractionated RT owing to relatively slow tumor growth [10,20,33,34]. While improved local control through increases in the fraction size was anticipated, such attempts raise concerns over potential exacerbation of fibrosis and stenosis in the trachea and bronchus, as well as radiation pneumonitis; however, comprehensive studies on this matter are currently insufficient [8]. Although patient numbers in the current study were insufficient, we may provide a clue that helps resolve this question. In the high-dose definitive RT group, patients were treated with two main dose schemes as follows: 60–66 Gy with hypofractionation (3.0 Gy/fx) and 70–74 Gy with conventional fractionation (2.0 Gy/fx) (Table 1). Considering the crude rates within the representative groups, local recurrence rates were 28.6 and 12.5%, while the incidence of Grade ≥3 toxicity was 21.4 and 12.5% in the hypofractionated and conventional fractionated groups, respectively. These findings suggest that using high-dose RT with conventional fractionation exceeding 70.0 Gy could potentially offer an effective and safe treatment approach. Additionally, this research calls into question the commonly held belief that the α/β ratio is 2, indicating a need for reconsideration.

As a strength, our study represents a comprehensive analysis of the largest number of patients with tracheobronchial ACC who received RT as a treatment with curative intent. Furthermore, this study provides valuable insights into dose-escalated RT for definitive aim and optimal dose scheme considering effectiveness and safety. However, considering the retrospective nature of this study and the limited number of patients, its capacity to establish statistical significance is moderate. Additionally, further research on the α/β ratio of ACC is needed.

## 5. Conclusions

In conclusion, our study includes the largest number of patients among published studies to date, illustrating the overall natural course of the disease and treatment outcomes. Our retrospective analysis of patients with tracheobronchial ACC demonstrated that high-dose RT exceeding 70.0 Gy in EQD2 with conventional fractionation may be effective and safe, especially for a definitive aim. Future investigations involving larger patient populations, extended follow-up durations, and the integration of advanced techniques are expected to provide a comprehensive understanding of the effectiveness and safety of dose escalation.

## Figures and Tables

**Table 1:** Dose scheme for the patients with tracheobronchial adenoid cystic carcinoma.

Dose Group	Dose Scheme	No. of Patients (%)
Definitive RT		
High-dose group	60 Gy/20fx	13 (59.1)
	74 Gy/37fx	7 (31.8)
	66 Gy/22fx	1 (4.5)
	70 Gy/35fx	1 (4.5)
Low-dose group	66 Gy/33fx	2 (50.0)
	45 Gy/15fx	1 (25.0)
	54 Gy/18fx	1 (25.0)
Postoperative RT		
High-dose group	74 Gy/27fx	3 (60.0)
	75 Gy/25fx	2 (40.0)
Low-dose group	60 Gy/30fx	13 (76.5)
	64 Gy/32fx	3 (17.6)
	57 Gy/29fx	1 (5.9)

RT, radiation therapy. Percentages are calculated within each dose group as a proportion.

**Table 2:** Pattern of failures among patients with tracheobronchial adenoid cystic carcinoma who received radiotherapy.

Outcome	No. of Patients (%)
Any recurrence	31 (64.6)
Any LP	7 (14.6)
Isolated LP	0 (0.0)
Any DM	31 (64.6)
Isolated DM	24 (50.0)
Both LP and DM	7 (14.6)
First site of failure	
Trachea or main bronchus	3 (6.3)
Lung	22 (45.8)
Bone	2 (4.2)
Liver	1 (2.1)
Mediastinal lymph node	3 (6.3)
Distant metastasis pattern	
Single organ	18 (37.5)
Multiple organs	13 (27.1)
Distant metastasis site	
Lung	27 (56.3)
Bone	7 (14.6)
Liver	6 (12.5)
Mediastinal lymph node	4 (8.4)
Brain	2 (4.2)
Others	1 (2.1)

LP, local progression; DM, distant metastasis.

**Table 3:** Characteristics of patients experiencing local progression.

Case No.	Primary Tumor Location	Dose Scheme	Dose Group	Time to LP (Months)	Survival Time (Months)
#1	Trachea to main bronchus	54 Gy/18fx	Low-dose	27.4	60.9
#2	Trachea to main bronchus	66 Gy/33fx	Low-dose	37.0	48.3
#3	Trachea	60 Gy/20fx	High-dose	62.3	104.7
#4	Trachea	60 Gy/20fx	High-dose	62.9	114.2
#5	Trachea	60 Gy/20fx	High-dose	44.4	51.8
#6	Trachea to main bronchus	60 Gy/20fx	High-dose	9.8	53.3
#7	Trachea to main bronchus	74 Gy/37fx	High-dose	55.7	115.1

RT, radiotherapy; fx, fraction; LP, local progression.

**Table 4:** Treatment outcomes of patients with tracheobronchial adenoid cystic carcinoma treated with definitive radiation therapy using a high-dose regimen.

First Author (Year)	No. of Patients	Radiotherapy Technique and Dose		Survival Outcomes (High-Dose vs. Low-Dose)	Complications with High-Dose Radiotherapy (%)
		High-Dose	Low-Dose		
OURS	48	Median EQD2, 75.0 Gy	Median EQD2, 67.5 Gy	5-yr FFLP (%): 88.6 vs. 33.3 5-yr OS (%): 79.0 vs. 66.7	Tracheal stenosis, G3 (19.0%)
Retrospective study					
Je (2017) [15]	9 ^a^	EBRT + BT boost, median 77.1 Gy (range, 70.8–80.0 Gy)	EBRT, median 66.0 Gy (range, 66.0–70.2 Gy)	5-yr LPFS (%): 100.0 vs. 0.0 5-yr OS (%): 83.3 vs. 33.3	Tracheal stenosis, G3 (16.7%)
Dracham (2022) [20]	12 ^b^	90.0 Gy	54.0–65.5 Gy	5-yr LRFS (%): 75.0 vs. 16.7	No Grade ≥3 toxicities
Chen (2021) [23]	7 ^c^	CIRT, ≥72.6–85.8 GyE (3.3 GyE/fx)	(-)	(-)	No Grade ≥3 toxicities
Case-report					
Millar (2012) [6]	2	80.0 Gy/40fx (2.0 Gy/fx)	(-)	NED for 6 years and 11 months, respectively	Chest pain, G1 (1 patient)
Verma (2018) [25]	5 ^d^	Proton 80.0 GyE/40fx (2.0 GyE/fx)	(-)	NED for 42 months	Esophagitis, G1 Dermatitis, G1 Tracheal stenosis, G3
Pawlewicz (2018) [26]	1	75.9 Gy/66fx (bid) (1.15 Gy/fx)	(-)	NED for 3 years	N/A
Spinelli (2019) [24]	1	70.0 Gy/35fx (2.0 Gy/fx)	(-)	NED for 1 year	No complications
Piorek (2021) [22]	1	EBRT + BT boost, 64.0 Gy/32fx (2.0 Gy/fx) + 10.0 Gy/2fx	(-)	NED for 8 years	Esophagitis, Grade 2 No late complications
Wu (2021) [21]	1	76.0 Gy/38fx (2.0 Gy/fx)	(-)	NED for 5 years	Esophagitis, Grade 2 Dysphagia, Grade 1

EBRT, external beam radiotherapy; FFLP, freedom from local progression; OS, overall survival; fx, fraction; BT, brachytherapy; LPFS, local progression-free survival; PFS, progression-free survival; LRFS, local recurrence-free survival; CIRT, carbon ion radiotherapy; NED; no evidence of disease; RT, radiotherapy; yr, year. ^a^ Twenty-two individuals were included in the overall analysis, with nine of them receiving definitive radiation therapy. The table includes only the outcomes of these patients. ^b^ Nineteen individuals were included in the overall analysis, with 12 patients treated with definitive RT and analyzed for survival outcomes by doses, of whom three patients were treated with high-dose RT. ^c^ Eighteen individuals were included in the overall analysis, with seven patients treated with high-dose CIRT. ^d^ Among five patients, only one was treated with high-dose proton radiotherapy.

## Data Availability

The data supporting this study’s findings are available on request from the corresponding author.

## Supplementary Materials

The following supporting information can be downloaded at: https://www.mdpi.com/article/10.3390/cancers16112127/s1, Table S1. Patient characteristics (n = 48); Table S2. Subsequent treatment after treatment failure; Table S3. Univariate analysis of clinical factors affecting freedom from local progression; Table S4. Univariate and multivariate analysis of clinical factors affecting overall survival; Table S5. Patient characteristics by the aim of radiotherapy and dose group; Table S6. Pattern of failures by the aim of radiotherapy and dose group; Table S7. Patients with Grade 3 toxicities in definitive radiation therapy group.
