# Supplementary material for: Dose-Escalated Radiotherapy for Primary Tracheobronchial Adenoid Cystic Carcinoma
Source: Cancers (Basel). 2024 Jun 3;16(11):2127. doi: 10.3390/cancers16112127 (PMC11171223; doi:10.3390/cancers16112127)
Supplement: Supplementary file 1 [file cancers-16-02127-s001.zip › cancers-3002646-supplementary.pdf]

Table S1. Patient characteristics (n=48)

Characteristics	No. of patients (%)
Median age, year, [range]	50 [24–76]
Sex	
Male	23 (47.9)
Female	25 (52.1)
Smoking status	
Never smoker	27 (56.3)
(Ex-) Smoker	21 (43.8)
Tumor size, cm, [mean ± SD]	2.5 ± 0.8
Clinical T stage	
T1	11 (22.9)
T2	14 (29.2)
T3	23 (47.9)
Clinical N stage	
N0	40 (83.3)
N1	8 (16.7)
Aim of RT	
Definitive RT	26 (54.2)
Postoperative RT	22 (45.8)
Extent of resection	
No operation	26 (54.2)
R0 resection	6 (12.5)
R1 resection	14 (29.2)
R2 resection	2 (4.2)
Stent insertion[a)]	
Yes	9 (18.8)
No	39 (81.3)
Median EQD2, Gy, [range]	74.0 [56.3–83.5]
Median dose per fx, Gy, [range]	2.0 [1.8–3.0]

SD, standard deviation; RT, radiotherapy; EQD2, equivalent dose at 2.0 Gy; fx, fraction.

[a)]Stent insertion before radiotherapy.

Table S2. Subsequent treatment after treatment failure

Failure pattern	Subsequent treatment after failure
Local progression	Stent insertion (n=3)
	Bronchoscopic resection (n=2)
	Observation & Conservative treatment (n=2)
Distant metastasis	
Lung	Observation & Conservative treatment (n=18)
	Chemotherapy (n=5)
	Surgical resection (n=3)
	RT (n=1)
Bone	RT (n=4)
	Observation (n=2)
	Chemotherapy (n=1)
Liver	Chemotherapy (n=3)
	Observation & Conservative treatment (n=2)
	Surgical resection (n=1)
Mediastinal lymph node	Observation & Conservative treatment (n=2)
	Chemotherapy (n=1)
	RT (n=1)
Brain	GKS (n=2)
Others	Observation & Conservative treatment (n=1)

RT, radiotherapy; GKS, gamma knife surgery.

Table S3. Univariate analysis of clinical factors affecting freedom from local progression

**Variables**	**HR (95% CI)**	**p**	**reference**
Age	1.00 (0.93–1.08)	0.938	[continuous]
Sex, Male	0.70 (0.11–4.61)	0.709	Female
MB involvement, Yes	2.54 (0.49–13.12)	0.264	No
Tumor size	0.78 (0.24–2.60)	0.691	[continuous]
T stage, T2-3[a)]	-	-	T1
N stage, N1	1.79 (0.20–16.10)	0.602	N0
Aim of RT, Definitive[a)]	-	-	Postoperative
RT dose (EQD2, Gy)	1.08 (0.95–1.24)	0.246	[continuous]
RT dose, High-dose[a)]	3.10 (0.60–16.14)	0.179	Low-dose
Resection, R1-2[a)]	-	-	R0
Stent insertion[b)], Yes	7.21 (0.65–79.58)	0.107	No

HR, hazard ratio; CI, confidential interval; MB, main bronchus; RT, radiotherapy; EQD2, equivalent dose at 2.0 Gy.

[a)]HR calculation was not feasible for this variable due to the presence of a group with no local progresion.

[b)]Stent insertion before radiotherapy.

Table S4. Univariate and multivariate analysis of clinical factors affecting overall survival

Variables	Univariate analysis		Multivariate analysis		Reference
	HR (95% CI)	p	HR (95% CI)	p	
Age	1.21 (0.99–1.07)	0.184	-	-	[continuous]
Sex, Male	0.99 (0.39–2.50)	0.982	-	-	Female
MB involvement, Yes	2.32 (0.88–6.13)	0.090	2.00 (0.72–5.41)	0.190	No
Tumor size	1.34 (0.77–2.33)	0.303	-	-	[continuous]
T stage, T2-3	5.26 (0.70–39.77)	0.108	-	-	T1
N stage, N1	3.98 (1.21–13.12)	0.023	8.66 (2.12–35.43)	0.003	N0
Aim of RT, Definitive	4.52 (1.50–13.64)	0.007	6.41 (1.79–23.00)	0.004	Postoperative
RT dose (EQD2, Gy)	1.01 (0.95–1.08)	0.711	-	-	[continuous]
RT dose, High-dose	1.24 (0.47–3.30)	0.668	-	-	Low-dose
Resection, R1-2	2.75 (0.56–13.41)	0.212	-	-	R0
Stent insertion[a)], Yes	2.46 (0.82–7.33)	0.108	1.56 (0.48–5.12)	0.464	No

HR, hazard ratio; CI, confidential interval; MB, main bronchus; RT, radiotherapy; EQD2, equivalent dose at 2.0 Gy.

[a)]Stent insertion before radiotherapy.

Table S5. Patient characteristics by the aim of radiotherapy and dose group.

Variables (n, (%))	Definitive RT			Postoperative RT		
	Low-dose (n = 4)	High-dose (n = 22)	p	Low-dose (n = 17)	High-dose (n = 5)	p
Age, median, year, [range]	62 [40–73]	52 [28–76]	0.453	44 [24–73]	46 [37–62]	0.520
Sex						
Male	3 (75.0)	12 (54.5)	0.614	6 (35.3)	4 (80.0)	0.135
Female	1 (25.0)	10 (45.5)		11 (64.7)	1 (20.0)	
Smoking status						
Never smoker	3 (75.0)	13 (59.1)	0.639	8 (47.1)	3 (60.0)	1.000
(Ex-) Smoker	1 (25.0)	9 (40.9)		9 (52.9)	2 (40.0)	
Tumor size, cm, [mean ± SD]	2.8±0.63	2.5± 0.76	0.444	2.7 ± 0.93	2.2 ± 0.33	0.614
Clinical T stage						
T1	0 (0.0)	4 (18.2)	0.238	3 (17.6)	0 (0.0)	0.491
T2	0 (0.0)	8 (36.4)		6 (35.3)	1 (20.0)	
T3	4 (100.0)	10 (45.5)		8 (47.1)	4 (80.0)	
Clinical N stage						
N0	2 (50.0)	21 (95.5)	0.052	13 (76.5)	4 (80.0)	1.000
N1	2 (50.0)	1 (4.5)		4 (23.5)	1 (20.0)	
Stent insertion[a]						
Yes	1 (25.0)	7 (31.8)	1.000	1 (5.9)	0 (0.0)	1.000
No	3 (75.0)	15 (68.2)		16 (94.1)	5 (100.0)	
EQD2, median, Gy, [range]	66.8 [56.3–69.3]	75.0 [70.0–82.5]	<0.001	60.0 [56.6–64.0]	74.0 [74.0–75.0]	<0.001
Daily dose, median, Gy, [range]	2.6 [2.0–3.0]	3.0 [2.0–3.0]	0.162	2.0 [1.8–2.0]	2.0 [2.0–3.0]	0.043

RT, radiotherapy; SD, standard deviation; EQD2, equivalent dose at 2.0 Gy; fx, fraction.

[a]Stent insertion before radiotherapy.

Table S6. Pattern of failures by the aim of radiotherapy and dose group.

Outcome (*n*, (%))	Definitive RT		Postoperative RT	
	**Low-dose (*n* = 4)**	**High-dose (*n* = 22)**	**Low-dose (*n* = 17)**	**High-dose (*n* = 5)**
Any recurrence	3 (75.0)	15 (68.2)	11 (64.7)	2 (40.0)
Any LP	2 (50.0)	5 (22.7)	0 (0.0)	0 (0.0)
Isolated LP	0 (0.0)	0 (0.0)	0 (0.0)	0 (0.0)
Any DM	3 (75.0)	15 (68.2)	11 (64.7)	2 (40.0)
Isolated DM	1 (25.0)	10 (45.5)	11 (64.7)	2 (40.0)
Both LP and DM	2 (50.0)	5 (22.7)	0 (0.0)	0 (0.0)
First site of failure				
Trachea or main bronchus	1 (25.0)	2 (9.1)	0 (0.0)	0 (0.0)
Lung	2 (50.0)	11 (50.0)	7 (41.2)	2 (40.0)
Bone	0 (0.0)	1 (4.5)	1 (5.9)	0 (0.0)
Liver	0 (0.0)	0 (0.0)	1 (5.9)	0 (0.0)
Mediastinal lymph node	0 (0.0)	1 (4.5)	2 (11.8)	0 (0.0)
Distant metastasis pattern				
Single organ	1 (25.0)	10 (45.5)	6 (35.3)	1 (20.0)
Multiple organs	2 (50.0)	5 (22.7)	5 (29.4)	1 (20.0)
Distant metastasis site				
Lung	3 (75.0)	13 (59.1)	9 (52.9)	2 (40.0)
Bone	2 (50.0)	3 (13.6)	3 (17.6)	0 (0.0)
Liver	0 (0.0)	3 (13.6)	2 (11.8)	1 (20.0)
Mediastinal lymph node	0 (0.0)	1 (4.5)	3 (17.6)	0 (0.0)
Brain	0 (0.0)	1 (4.5)	1 (5.9)	0 (0.0)
Others	0 (0.0)	0 (0.0)	1 (5.9)	0 (0.0)

RT, radiotherapy; LP, local progression; DM, distant metastasis.

Table S7. Patients with grade 3 toxicities in definitive radiation therapy group

Case no.	Involvement of MB	Dose scheme	Daily dose	Dose group
#1	No	60 Gy/20fx	3 Gy	High-dose group
#2	Yes	60 Gy/20fx	3 Gy	High-dose group
#3	Yes	60 Gy/20fx	3 Gy	High-dose group
#4	Yes	74 Gy/27fx	2 Gy	High-dose group

MB, Main bronchus.
